# First in human neochordae retensioning for recurrence of mitral regurgitation after neochord procedure

**DOI:** 10.3389/fcvm.2023.1120323

**Published:** 2023-05-15

**Authors:** Alessandro Fiocco, Demetrio Pittarello, Augusto D’Onofrio, Florinda Mastro, Gino Gerosa, Andrea Colli

**Affiliations:** ^1^ Cardiac Surgery Unit, Department of Surgical, Medical and Molecular Pathology and Critical Care Medicine, University of Pisa, Pisa, Italy; ^2^ Cardiac Surgery Unit, Department of Cardiac, Thoracic and Vascular Sciences and Public Health, University of Padua, Padua, Italy

**Keywords:** mitral valve, mitral valve repair, retensioning, Neochord procedure, microinvasive

## Abstract

The Neochord procedure is a viable option to treat degenerative mitral valve regurgitation in selected patients. Left ventricle reverse remodeling can cause neochord-relative elongation and reprolapse of the treated leaflet, leading to failure. We present a clinical case of extensive ventricle reverse remodeling after neochord implantation and the first-in-man off-pump surgical retensioning of the previously implanted artificial chords.

## Introduction

The Neochord procedure is recognized as a possible therapeutic option to treat specific subsets of patients presenting degenerative mitral valve regurgitation (DMR) (1–4). Although different causes of failure have been reported in clinical practice, they can be categorized into three groups: recurrence of the treated leaflet prolapse, occurrence of pseudoprolapse of the untreated leaflet, and true prolapse of the untreated leaflet. Causes of recurrence of prolapse are neochord insertion tearing on the leaflet free edge or neochord-relative elongation (NCRE) consequent to left ventricle reverse remodeling (LVRR) (5). The cause of pseudoprolapse of the untreated leaflet is the excessive intraoperative overtensioning of the implanted neochords. Causes of new prolapse occurrence are iatrogenic damage of the subvalvular apparatus due to inadequate intraventricular navigation of the device during neochord placement or postoperative native chordal rupture due to continuous friction between artificial and native chords (5).

In case of mitral regurgitation (MR) recurrence, depending on the causing mechanism, the patient can undergo new conventional open-heart mitral valve (MV) repair, Neochord procedure reintervention, or, in selected cases, Neochord retensioning procedure.

We aim to report the surgical description of how to perform an off-pump, mini-invasive Neochord retensioning procedure in case of MR recurrence caused by NCRE of the implanted chords due to LVRR.

## Case description

A 66-year-old man was referred to our institution to be evaluated for elective correction of severe MR. The patient was also affected by grade 1 obesity (32.6 kg/m^2^), arterial hypertension, dyslipidemia, chronic obstructive pulmonary disease, and paroxysmal atrial fibrillation. He only presented with exertional dyspnea (NYHA IIb/III). Physical examination reported arrhythmic heart beats and systolic heart murmur, irradiated to the axillary line. Arterial blood pressure was controlled with Ca2-antagonist and diuretics.

He underwent transesophageal echocardiography (TEE), revealing isolated P2 scallop flail and severe degenerative MR.

He was screened and deemed eligible for the Neochord procedure.

Therefore, he underwent the surgery, in the standard fashion, that is step-by-step described elsewhere (6). The patient underwent the implantation of three pairs of neochords on the posterior mitral leaflet (PML), with residual trivial MR and no intraprocedural complications. He was extubated in the operative room (OR), transferred to the intensive care unit, and moved to the ward on postoperative day (POD) 1.

**Figure 1:**
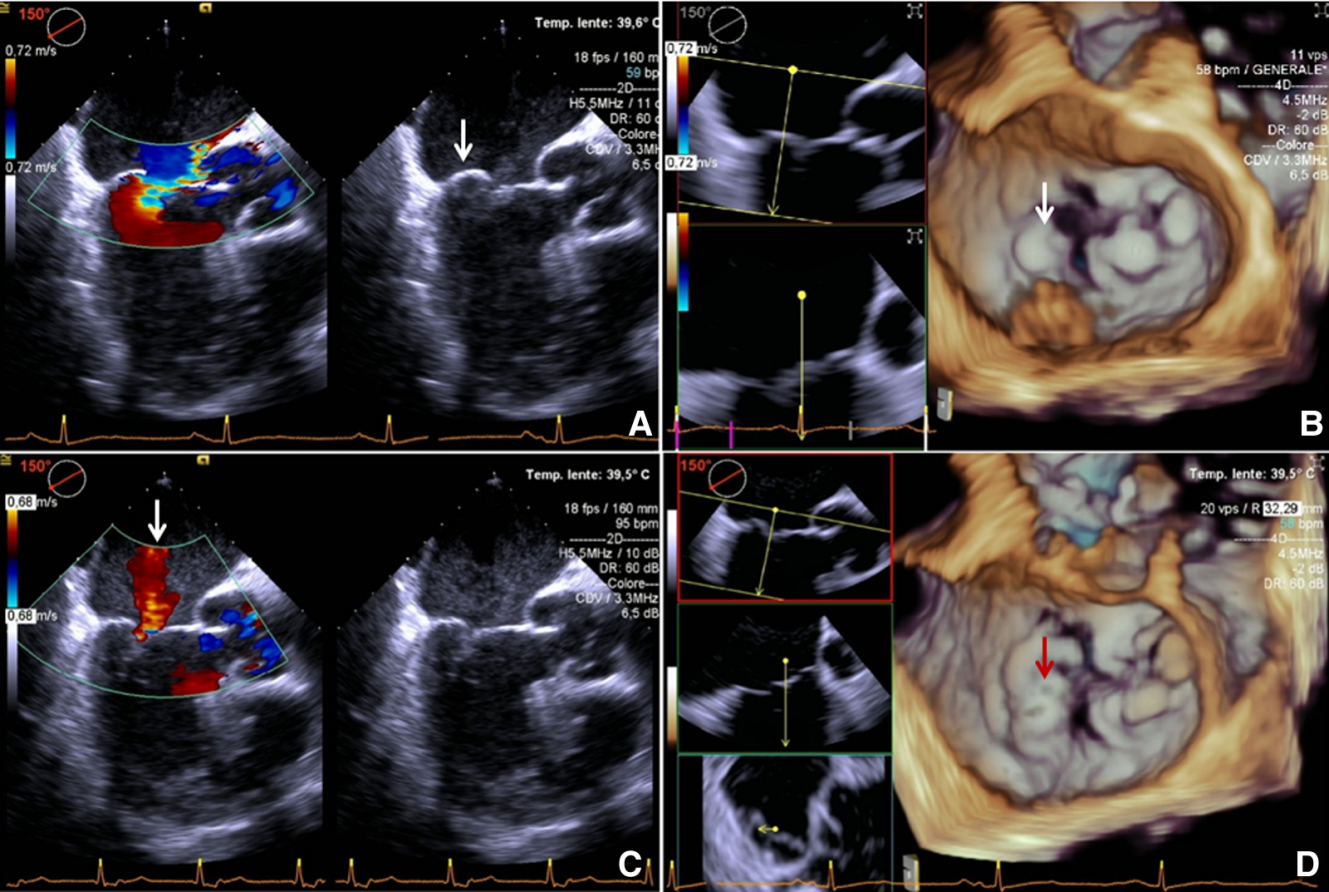
Intraoperative transesophageal echocardiography. (**A,B**): before retensioning procedure 2D + color Doppler and 3D; eccentric regurgitation jet directed to AML and reprolapsing PML (arrow). (**C,D**): after retensioning procedure 2D + color Doppler and 3D: (**C**) central postprocedural mild MR (arrow) is showed. (**D**) Mild residual PML prolapse is highlighted (arrow) and a good coaptation between AML and PML is reached. AML, anterior mitral leaflet; MR, mitral regurgitation; PML, posterior mitral leaflet.

On POD 3, physical examination revealed 3/6 systolic heart murmur and postoperative transthoracic echocardiography (TTE) showed severe MR recurrence. TEE confirmed these findings, in the absence of native/artificial chordal rupture and in the presence of significant LVRR, causing NCRE and reprolapse of the treated leaflet (Figures 1A,B). Preoperative end-diastolic volume index (iLVEDV) was 98 ml/m^2^, and postoperative iLVEDV was 81 ml/m^2^.

Therefore, we decided to surgically recorrect the MR.

## Technique

**Figure 2:**
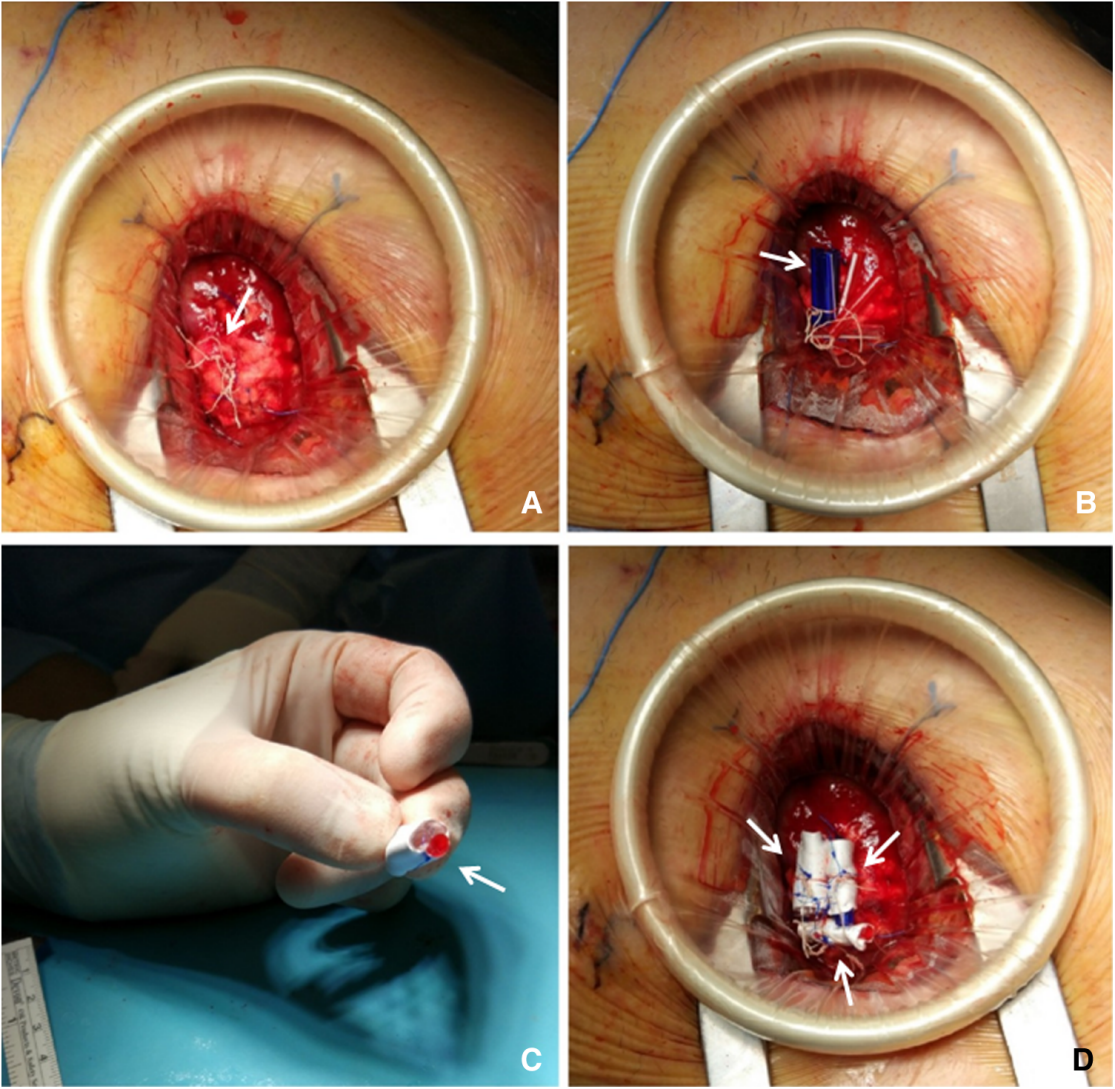
(**A**) Apex re-exposure through previous left mini-thoracotomy reopening: Identification of the chordal loops sprouting from the Teflon pledget (arrow) corresponding to the ventricular access site. (**B**) Samples sizing: temporary samples made of tourniquet tubing pieces (arrow) are positioned inside the loops, between the knots and the Teflon pledget. (**C**) Defining final samples: once the desired shape is set, the tourniquet pieces are fixed together with a 4-0 prolene suture and then covered with a Gore-Tex vascular prosthesis (arrow). (**D**) Samples fixation: definitely samples (arrows) are inserted inside the chordal loop and then fixed by suturing the vascular prosthesis to the corresponding loop with a 4-0 prolene suture.

The procedure was performed in a standard OR, under general anesthesia. First, the left ventricular (LV) apex was reapproached through the already existing left mini-thoracotomy. Once the implanted neochords were identified (Figure 2A), they were manually retensioned under real-time TEE guidance. Temporary samples were interposed below the external loop to shorten the internal portion of neochords up to the desired length. These samples were built by a variable number of tourniquet tubing pieces fixed together (Figure 2B); the correct size was defined by increasing/decreasing the thickness of each sample by adding/removing tourniquet tubing pieces. The final length of the neochords was established once adequate coaptation was restored and optimal correction of MR was obtained. Each final sample (Figure 2C) was then inserted inside a Gore-Tex 8 mm caliber vascular prosthesis. We built three samples (one for each chordal loop) and fixed them to the neochordal loops with sutures (Figure 2D). The final result was residual mild central MR (Figures 1C,D).

## Discussion

In the Paduan Single Center experience, Neochord procedure failure was observed in 9% at 1-year follow-up (7). Most of the events occurred during the early adoption phase of the technique, being mostly related to the absence of extensive clinical experience with the procedure and consequent lack of standardized procedural steps and precise patient selection criteria.

Reprolapse of the treated leaflet due to relative elongation of previously implanted neochords has been already described (5). For this reason, a slight overtension of the neochords is always suggested in order to prevent the occurrence of the abovementioned mechanism of procedure failure. The adequate neochord overtension achievement is based on real-time TEE guidance and established when a satisfactory coaptation point is gained with no significant anterior mitral leaflet pseudoprolapse. In our experience, we have observed that the overtensioning disappears after few months, once a slight LVRR occurs (1, 3), maintaining a correct MV leaflet coaptation.

In the reported case, preoperative iLVEDV was 98 ml/m^2^ and postoperative iLVEDV was 81 ml/m^2^, a so-called “extensive LVRR occurrence.”

The degree of LVRR is significantly variable, depending on non-predictable single-patient features that have not been clearly identified yet (8). Acute LVRR has already been reported after the Neochord procedure (9), but the quantification and prediction ofthe phenomenon are a current issue. Therefore, the extent of theovertensioning is actually a patient-tailored and surgeon eye-ball decision, being not performed applying quantitative echocardiographic measurements. So, the possibility of an unpredictable excessive volume reduction with consequent reprolapse of the treated leaflet must be considered and the patient must be warned about it, during informed consent acquisition.

In the reported case, residual MR was trivial after the first procedure. On postoperative day 3, recurrence of severe MR was reported (Figures 2A,B). Therefore, we decided to undergo a new surgery.

The first issue that we faced was how to perform valuable retensioning in a feasible and efficient way. It appeared to be very challenging to untie the previous knots and tie them again to modify the neochord length. For this reason, we propose for the first time a surgical procedure to obtain leaflet retensioning by interposing samples whose sizes have been intraoperatively decided under real-time TEE guidance (4). Once the right size has been decided, the samples are covered with a biocompatible Gore-Tex vascular prosthesis to minimize the risk of foreign body granuloma formation and also to increase tourniquet pieces stability and sample adhesion.

Based on the minimal invasiveness of the present retensioning procedure, and the anatomophysiological consideration on which it is based, we propose it to be considered before conversion to conventional on-pump surgery in case on failing Neochord procedure, in selected patients. To simplify the re-exposure of the LV access site, we have recently started to employ on a routine base reabsorbable biological sealant (Coseal, Baxter Healthcare, Hayward Corporation, CA, United States), reducing the formation of pericardial adhesions.

## Limitations and future perspectives

LVRR and subsequent NCRE represent a major issue to be investigated, being one of the causes of procedural failure. This involves not only transapical Neochord procedure but actually will be a crucial matter dealing also with next generation of transcatheter transseptal chordal implantation devices, whose introduction in the clinical practice will occur in the very next future.

The neochord length was not measured in the present case report. We did not perform any CT scan that could have been useful to describe the initial and post-retensioning length. The tensioning, during the first procedure, and the retensioning (second procedure) were not preoperatively predicted, since the current optimal way to achieve the desired length is by means of intraprocedural real-time TEE guidance, shortening the implanted neochords until MR is corrected.

Therefore, future perspectives should be to achieve better patient selection criteria, investigating predisposal factors to LVRR, to predict cases of abundant volume reduction after MR correction, especially in case of artificial chordal implantation procedures.

A new engaging perspective will be to investigate the future development of a new-generation transcatheter device that can allow for postprocedural neochord shortening, in case of extensive LVRR and subsequent NCRE.

## Conclusion

MR recurrence after the Neochord procedure could be caused by different mechanisms. This report could contribute to make a differential diagnosis of the different mechanisms of failure.

One of this could be the physiopathological phenomenon of extensive LVRR after mitral regurgitation correction, causing NCRE. In this subset, we propose to consider the described off-pump, mini-invasive retensioning procedure rather than conventional on-pump re-repair surgery in selected patients.

## Data Availability

The original contributions presented in the study are included in the article, further inquiries can be directed to the corresponding author.
